# Phylogenetic shifts of bacterioplankton community composition along the Pearl Estuary: the potential impact of hypoxia and nutrients

**DOI:** 10.3389/fmicb.2015.00064

**Published:** 2015-02-10

**Authors:** Jiwen Liu, Bingbing Fu, Hongmei Yang, Meixun Zhao, Biyan He, Xiao-Hua Zhang

**Affiliations:** ^1^College of Marine Life Sciences, Ocean University of ChinaQingdao, China; ^2^Key Laboratory of Marine Chemistry Theory and Technology, Ministry of Education, Ocean University of ChinaQingdao, China; ^3^State Key Laboratory of Marine Environmental Science, Xiamen UniversityXiamen, China; ^4^School of Bioengineering, Jimei UniversityXiamen, China

**Keywords:** bacterial community, hypoxia, Pearl Estuary, 16S rRNA gene, 454 pyrosequencing

## Abstract

The significance of salinity in shaping bacterial communities dwelling in estuarine areas has been well documented. However, the influences of other environmental factors such as dissolved oxygen and nutrients in determining distribution patterns of both individual taxa and bacterial communities inhabited local estuarine regions remain elusive. Here, bacterioplankton community structures of surface and bottom waters from eight sites along the Pearl Estuary were characterized with 16S rRNA gene pyrosequencing. The results showed significant differences of bacterioplankton community between freshwater and saltwater sites, and further between surface and bottom waters of saltwater sites. *Synechococcus* dominated the surface water of saltwater sites while *Oceanospirillales*, SAR11 and SAR406 were prevalent in the bottom water. *Betaproteobacteria* was abundant in freshwater sites, with no significant difference between water layers. Occurrence of phylogenetic shifts in taxa affiliated to the same clade was also detected. Dissolved oxygen explained most of the bacterial community variation in the redundancy analysis targeting only freshwater sites, whereas nutrients and salinity explained most of the variation across all samples in the Pearl Estuary. *Methylophilales* (mainly PE2 clade) was positively correlated to dissolved oxygen, whereas *Rhodocyclales* (mainly R.12up clade) was negatively correlated. Moreover, high nutrient inputs to the freshwater area of the Pearl Estuary have shifted the bacterial communities toward copiotrophic groups, such as *Sphingomonadales*. The present study demonstrated that the overall nutrients and freshwater hypoxia play important roles in determining bacterioplankton compositions and provided insights into the potential ecological roles of specific taxa in estuarine environments.

## Introduction

Bacterioplankton is a key component of the microbial food web by means of carbon delivery and it plays significant roles in biogeochemical cycles (Azam et al., 1983). In estuarine systems, which were subject to intensive anthropogenic disturbance, microbes take significant parts in decomposition of autochthonous and allochthonous organic matter as well as removal of contaminants. The strong natural and anthropogenic gradients in estuaries make them ideal niches for investigating the response of microbes to various environmental forces (Telesh and Khlebovich, 2010). For example, the natural salinity gradient is considered as a major factor in structuring microbial community compositions in estuarine systems (Lozupone and Knight, 2007; Fortunato et al., 2012).

The increasing discharge of nutrients and pollutants into estuaries deteriorates the water quality severely, resulting in algal bloom and subsequent hypoxia. Dissolved oxygen (DO) has been demonstrated to be capable of altering bacterial communities in many marine and fresh water systems (Stevens and Ulloa, 2008; Zaikova et al., 2010; Li et al., 2012). The low DO concentration could compress the habitat available to aerobic organisms and alter the food chain structure (Rabalais et al., 2010), resulting in changes of biogeochemical cycles (e.g., Naqvi et al., 2000). The high nutrient inputs from anthropogenic activities could also alter the taxonomic composition of bacterial community (Simonato et al., 2010). However, little is known about the impacts of hypoxia and nutrients on the bacterioplankton community in estuarine systems (Crump et al., 2007), especially in freshwater areas. Moreover, effects of other environmental factors likely contributing significantly to bacterial variations were previously neglected in part due to the overwhelming influence of salinity.

The significance of microbes in regulating biogeochemical processes has intrigued tremendous interests in their compositions and distribution patterns. Over the past decade, surveys of bacterial community structure in various estuaries such as the Chesapeake Bay (Bouvier and Del Giorgio, 2002), Delaware Estuary (Kirchman et al., 2005; Campbell and Kirchman, 2013), Columbia River estuary (Fortunato and Crump, 2011) and Pearl Estuary (Wu et al., 2004; Zhang et al., 2006) have been conducted, and most of their efforts focused on variations in bacterial assemblages at relatively higher taxonomic levels such as phylum level. The lack of taxonomic resolution is a hurdle for exploring the specific taxa that contribute to the observed community variation (Fortunato et al., 2013). Moreover, some evidence of phylogenetic shift in clades within the same taxa has emerged from studies on microbial diversity using the deep sequencing method (Herlemann et al., 2011; Fortunato et al., 2012). The microbial distribution patterns at a finer resolution are of particular importance to uncover the actual microbial response to environmental changes. Estuarine systems with strong environmental gradients are therefore excellent candidates to elucidate the microbial distribution patterns of high precision. The Pearl River ranks 2nd and 13th in terms of discharge volume in China and among the world, respectively, with 20% of the discharge occurring during the dry season and 80% during the wet season (Zhao, 1990). Hypoxia (DO < 2 mg L^−1^) has been reported in the Pearl River (Dai et al., 2006), similar to other highly impacted estuaries such as the Scheldt Estuary and Seine Estuary (Frankignoulle et al., 1996; Garnier et al., 2001). The Lingdingyang Bay is the largest sub-estuary of the Pearl Estuary, which receives discharges from four eastern outlets (Humen, Jiaomen, Hongqimen, and Hengmen) (PRWRC/PRRCC, 1991). The broad width of the lower Pearl Estuary (~60 km) facilitates penetration of seawater into the bottom estuary thus causing strong vertical stratification especially in the wet season (Dong et al., 2004). However, microbial assemblages inhabited these two water masses are still unclear. In this study, bacterioplankton communities of 16 surface and bottom water samples from three freshwater and five saltwater sites were investigated using 16S amplicon pyrosequencing and flow cytometry. Statistical analyses were performed to evaluate the effects of nutrients and hypoxia on bacterial communities and specific taxa. Our study confirms the presence of two different bacterioplankton communities in the lower Pearl Estuary and provides information of resolved community variations in response to different environmental factors.

## Materials and methods

### Site description and sampling

**Figure 1:**
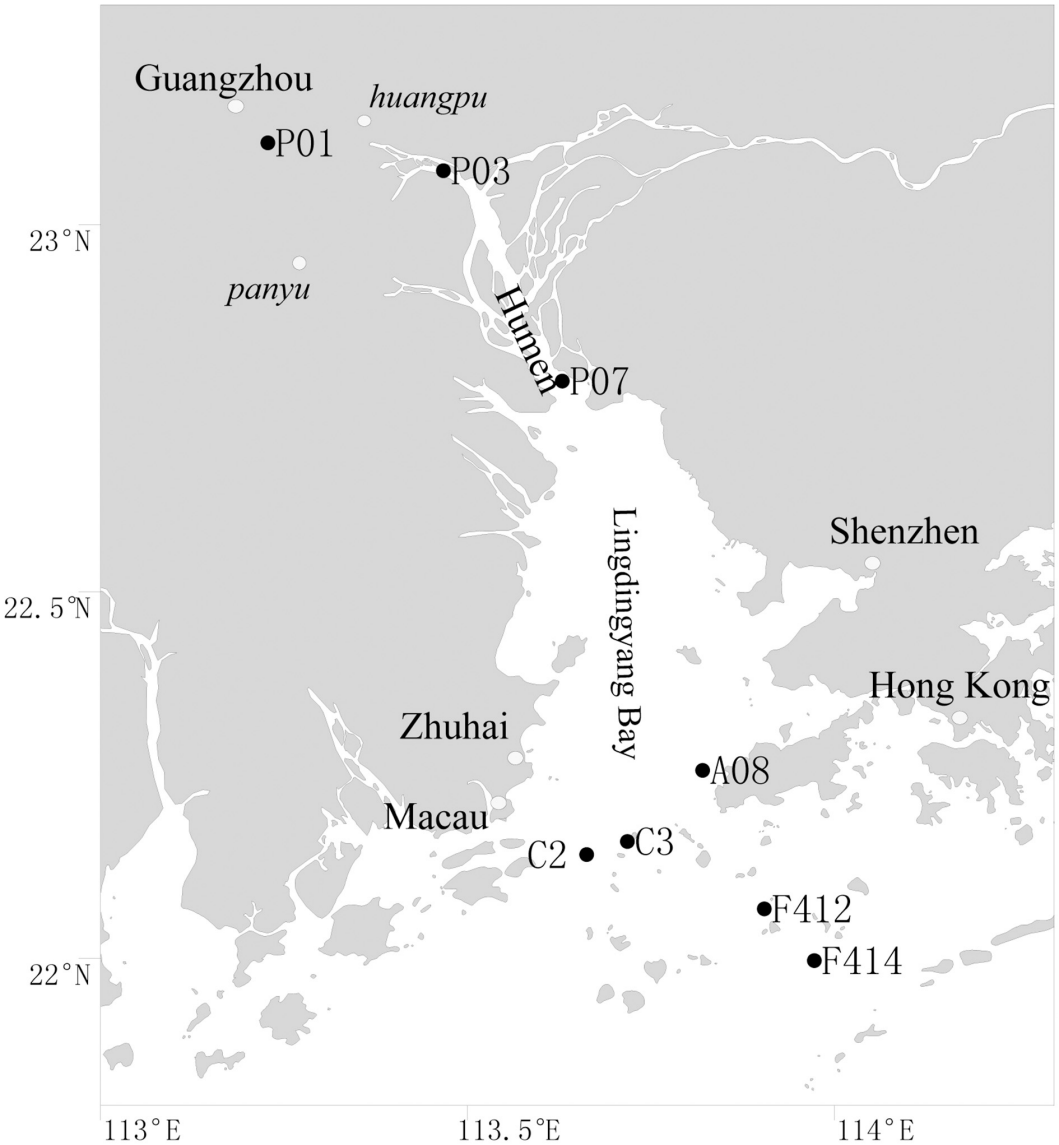
**Location of sampling sites in the Pearl Estuary**.

Surface (1 m depth) and bottom (1–2 m above sediment) waters were collected along the salinity gradient of the Pearl Estuary from 16 July to 12 August, 2012 using a Sealogger CTD (SBE 25, Sea-Bird Co.) rosette water sampler (Liu et al., 2014). A total of 16 water samples from eight sites, including P01, P03, and P07, located in the freshwater part of the estuary, C2, C3, and A08, located in the mesohaline region, and F412 and F414, located in the polyhaline region, were collected (Figure 1). One liter of water samples was pre-filtered through 3-μm-pore size filters to remove large organisms and particles, and free-living bacterioplankton cells were collected through 0.22-μm polycarbonate membranes (Millipore Corporation, Billerica, MA, USA). Filters were frozen at −80°C until DNA extraction. Water chemistries such as salinity, temperature, pH, turbidity and DO were monitored with the CTD. Samples for nutrients (NO^−^_2_, NO^−^_3_, NH^+^_4_, and PO^3−^_4_) were filtered with 0.45-μm cellulose acetate membranes and analyzed on deck with a QuAAtro (Bran-Lube) except for NO^−^_3_, which was analyzed in land-based laboratory at Xiamen University with spectrophotometric and colorimetric methods using an AA3 Auto-Analyzer (Technicon) (Dai et al., 2008; Han et al., 2012; Liu et al., 2014).

### Abundance of picoplankton

The collected samples were immediately mixed with paraformaldehyde (Sigma-Aldrich, St. Louis, MO, USA; final concentration 2%, v/v) for 1 h in the dark at room temperature, and then stored in liquid nitrogen onboard. The abundances of *Synechococcus*, picoeukaryotes and heterotrophic bacteria were measured by flow cytometry (BD FACSVantage SE, Becton, Dickinson, USA) (Marie et al., 1997).

### DNA extraction, PCR and 454 sequencing

DNA was extracted according to Yin et al. (2013) with an additional step to facilitate cell lysis by a Fast Prep-24 Homogenization System (MP Biomedicals, Irvine, California, USA). Amplification of bacterial 16S rRNA gene fragments was conducted using barcode and adaptor added primer 28F (5′-AGAGAGTTTGATCCTGGCTCAG-3′) and 519R (5′-TTACCGCGGCTGCTGGCAC-3′) (Wu et al., 2012). Barcode sequences were ligated to the sequencing primer during the process of primer synthesis, before PCR was performed. A 20 μl PCR reaction was performed in triplicate at the following condition: an initial denaturation at 95°C for 2 min, 25 cycles of 95°C for 30 s, 55°C for 30 s, and 72°C for 30 s, and then a final extension at 72°C for 5 min. The triplicate PCR products were pooled and purified using an AxyPrepDNA Gel Extraction Kit (Axygen, Hangzhou, China), and then quantified using a Quant-iT PicoGreen double-stranded DNA assay (Invitrogen, Carlsbad, CA). The amplicons from each reaction mixture were pooled in equimolar ratio based on concentration and subjected to emulsion PCR to generate amplicon libraries. Sequencing was carried out on a Roche Genome Sequencer FLX Titanium platform at Majorbio Bio-Pharm Technology Co., Ltd., Shanghai, China.

The 454 sequences have been deposited at the National Center for Biotechnology Information (NCBI) Short Read Archive database under accession number SRP019932.

### Sequence quality control and operational taxonomic unit (OTU) assignment

The raw reads were processed following the pipeline of Mothur (Schloss et al., 2009). All reads completely matching the barcodes were retained as well as reads with a maximum single mismatch to the primers. Reads were then trimmed by removing the sequencing adaptor, barcodes and primer sequences. These reads were further screened by using the following thresholds: (i) minimum average quality score of 25; (ii) minimum read length of 200 bp; (iii) sequences containing no ambiguous bases; and (iv) maximum homopolymers of 8 bp. Chimeric sequences were identified and removed using UCHIME Ref (Edgar et al., 2011). “Pre-cluster” (Huse et al., 2010) was further used to remove reads caused by sequencing error. Totally 86468 reads were left after quality control and were clustered into OTUs at a 3% distance level with the furthest neighbor algorithm. Taxonomy was assigned against the SILVA v111 ref database (http://www.arb-silva.de) at a minimum support threshold of 80%.

### Statistical analyses

The distribution and abundance matrix of OTUs was randomly resampled using “daisychopper.pl” (Gilbert et al., 2009) to equalize sampling efforts. After normalizing, 4354 sequences were left for each sample. Alpha diversity measures including richness estimator Chao 1 (Chao and Bunge, 2002), diversity index Shannon (Magurran, 1988) and Good's coverage (Good, 1953) were calculated at a 3% dissimilarity level in Mothur. For the beta diversity, a multi-sample similarity dendrogram was constructed according to the OTUs composition and abundance based on Bray-Curtis dissimilarity. Pairwise analyses of similarities (ANOSIM) of bacterioplankton communities were calculated in PRIMER 5 (Plymouth Marine Laboratory, West Hoe, Plymouth, UK). A ternary plot was generated in R software (RDC Team, 2008) to compare the community composition of different groups at the genus level. Redundancy analysis (RDA) with Monte Carlo test was performed to calculate the relationship between bacterial clades and water properties at both order and genus levels, following the results of pretested detrended correspondence analysis using Canoco (Version 4.5, Microcomputer Power). A neighbor-joining phylogenetic tree comparing bacterial community compositions in different estuaries was constructed by MEGA 5 (Tamura et al., 2011).

## Results

### Environmental characterization

Considering the shallow water depth of the Pearl Estuary [Table S1 (Liu et al., 2014)], only surface and bottom water samples were collected, which were designated _S (surface) and _B (bottom), respectively in the following analysis. The freshwater and saltwater end members were separated by the Humen Outlet. The physicochemical attributes of waters from both freshwater and saltwater regions have been described in detail [Table S1 (Liu et al., 2014)]. Briefly, the freshwater sites had significantly lower levels of salinity, DO and pH, and higher levels of nutrients and Chl *a* than the saltwater sites. There were clear vertical environmental gradients in the saltwater area, with the surface water possessing a higher level of NO^−^_3_, and lower levels of turbidity and salinity compared with the bottom water. The DO content of the freshwater sites was extremely low (<1 mg L^−1^) except for P03 (>3 mg L^−1^), indicative of severe hypoxia.

### Abundance of picoplankton

The overall abundance of heterotrophic bacteria from flow cytometry decreased from the freshwater to saltwater sites and was positively correlated with nutrients (Figure S1). *Synechococcus* and picoeukaryotes displayed opposite trends and both of them peaked in surface waters of sites A08 and F412. While picoeukaryotes were positively correlated with nutrients, *Synechococcus* was negatively correlated with turbidity (Figure S1). In the freshwater sites, the abundances of all three groups were basically constant between water layers, whereas in the saltwater sites, higher abundances of all three groups were observed in the surface water than the bottom water.

### Richness and diversity estimators

Totally, 86468 reads, ranging from 4354 to 6504 in all samples, were obtained for further analyses. The average length of the obtained reads was 470 base pairs. After randomly resampling, a total of 5314 OTUs were assigned at the 3% dissimilarity threshold. Both of the rarified Chao1 and Shannon diversity indexes basically showed no remarkable differences (*P* > 0.05, Wilcoxon's rank test) across all samples with the exception of surface waters of sites F412 and F414, where lower estimators were observed (Table 1). This was further confirmed by the Shannon curves that plotted with rarefied data (Figure S2). The Good's coverage values at the 3% dissimilarity level were 84.4–94.3% among all samples indicating that the libraries could represent most of the species in the natural habitat.

**Table 1:** **The original and rarefied OTUs, Chao1 and Shannon diversity indexes of the 16 samples collected from the Pearl Estuary at a 0.03 distance level**.

**Sample**	**Reads**	**3% dissimilarity**						
		**OTU**	**Chao I**	**Shannon**	**Good's coverage (%)**	**Normalized OTU**	**Normalized Chao I**	**Normalized Shannon**
P01_S	6504	1328	2623	5.92	88.9	895	1632	5.67
P01_B	5798	1308	2266	6.04	88.4	963	1756	5.77
P03_S	5407	1000	2106	5.49	89.7	738	1404	5.22
P03_B	4616	917	1670	5.4	89.4	778	1356	5.22
P07_S	4552	1053	1872	5.93	88.1	928	1600	5.80
P07_B	4705	1281	2514	6.17	84.4	1069	2126	5.97
C2_S	4440	1013	2071	5.59	86.8	950	1916	5.52
C2_B	5473	1087	2127	5.75	89.1	818	1584	5.49
C3_S	4354	956	1834	5.67	87.9	956	1834	5.67
C3_B	6296	935	1626	5.51	92.9	661	1074	5.28
A08_S	5549	1076	1946	5.61	89.7	797	1472	5.33
A08_B	5584	825	1458	5.33	92.9	632	1032	5.12
F412_S	6452	712	1324	4.28	94.4	480	808	4.06
F412_B	5932	901	1612	5.26	92.3	650	1075	5.04
F414_S	5291	718	1363	4.84	93.0	552	910	4.64
F414_B	5515	829	1320	5.49	93.3	616	909	5.28

*_S and _B standed for the surface and bottom water, respectively*.

### Taxonomic assignment

**Figure 2:**
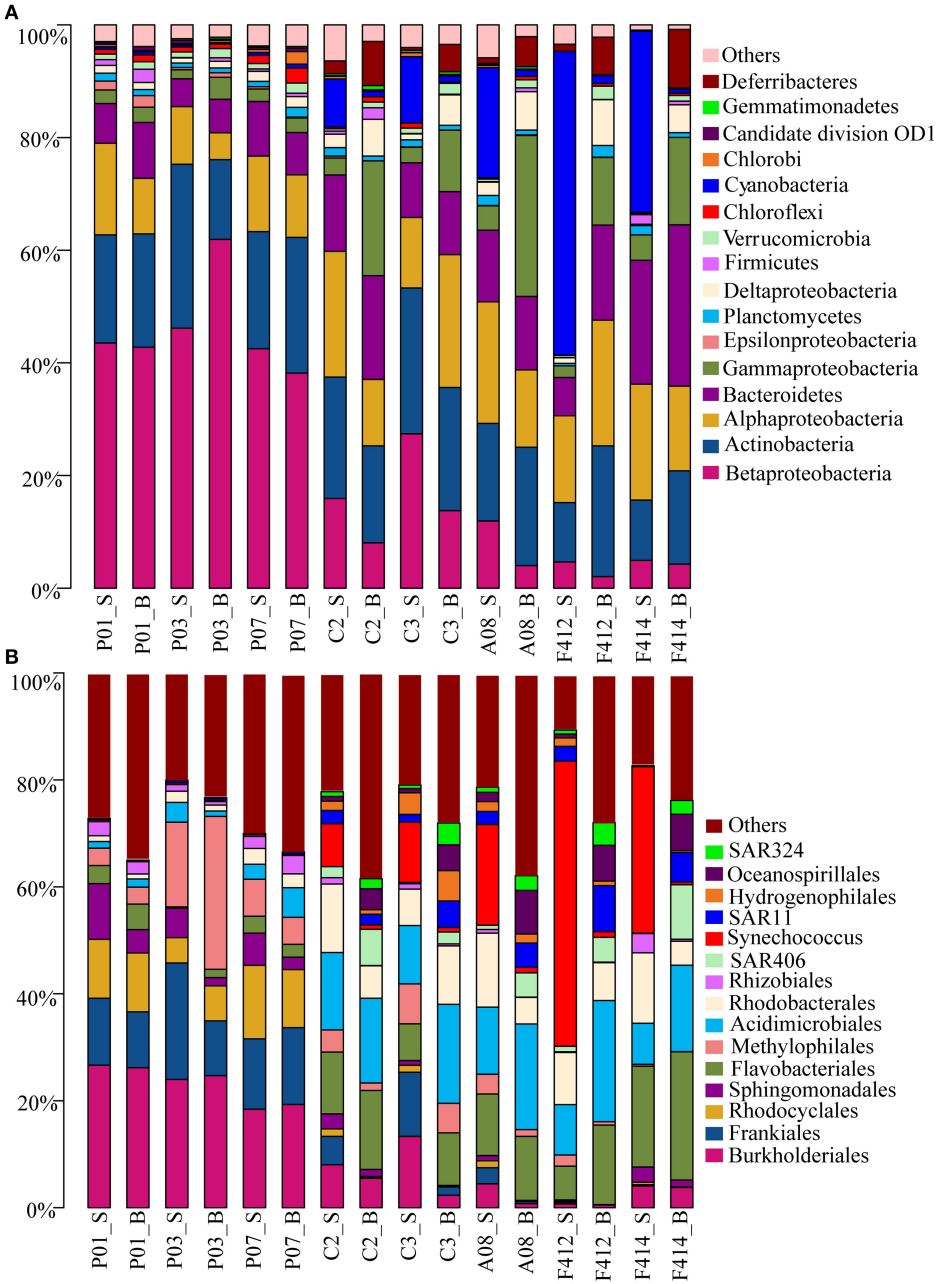
**Bacterial community compositions at different taxonomic levels across all samples**. **(A)** Relative abundances of the dominant bacterial phyla; **(B)** Relative abundances of phylotypes of the same phylum at deeper classifications (order-genus). _S and _B standed for the surface and bottom water, respectively.

Totally, 41 bacterial phyla were found. Significant variations in bacterioplankton communities at the phylum level (plus proteobacterial classes) were observed along the salinity gradient. While marked distinction in bacterial assemblages was observed between the two depths in the saltwater sites, no consistent difference was observed in the freshwater sites (Figure 2A).

*Betaproteobacteria* dominated the freshwater sites but its orders distributed differently along the estuary, with *Burkholderiales* (*r* = −0.937, *P* < 0.05; Pearson correlation coefficient with salinity) and *Rhodocyclales* (*r* = −0.851, *P* < 0.05), predominant in the freshwater sites whereas *Hydrogenophilales* in the saltwater sites. It was noticeable that the abundance of *Methylophilales* was markedly different among the three freshwater sites with a higher amount at site P03. *Synechococcus* of *Cyanobacteria* was the most predominant group in the surface water of saltwater sites, consolidating the result of flow cytometry, whereas *Oceanospirillales* of *Gammaproteobacteria*, SAR406 clade of *Deferribacteres* and SAR324 clade of *Deltaproteobacteria* were more prevalent in the bottom water.

There were slight increases of *Alphaproteobacteria* and *Bacteroidetes* from freshwater to saltwater samples with *Alphaproteobacteria* occupying both depths of the saltwater sites whereas *Bacteroidetes* was slightly higher in the bottom. Among *Alphaproteobacteria*, orders *Sphingomonadales* and *Rhizobiales* were more abundant in the freshwater sites whereas *Rhodobacterales* dominated the saltwater sites. In addition, a small amount of SAR11 clade was detected in the bottom water of saltwater sites. Within *Bacteroidetes*, the abundance of *Flavobacteriales* was significantly higher in saltwater samples than freshwater ones. *Actinobacteria* was almost consistent across all samples, within which, *Frankiales* and *Acidimicrobiales* dominated the freshwater and saltwater sites, respectively, and the abundance of *Acidimicrobiales* was higher in the bottom water (Figure 2B).

**Figure 3:**
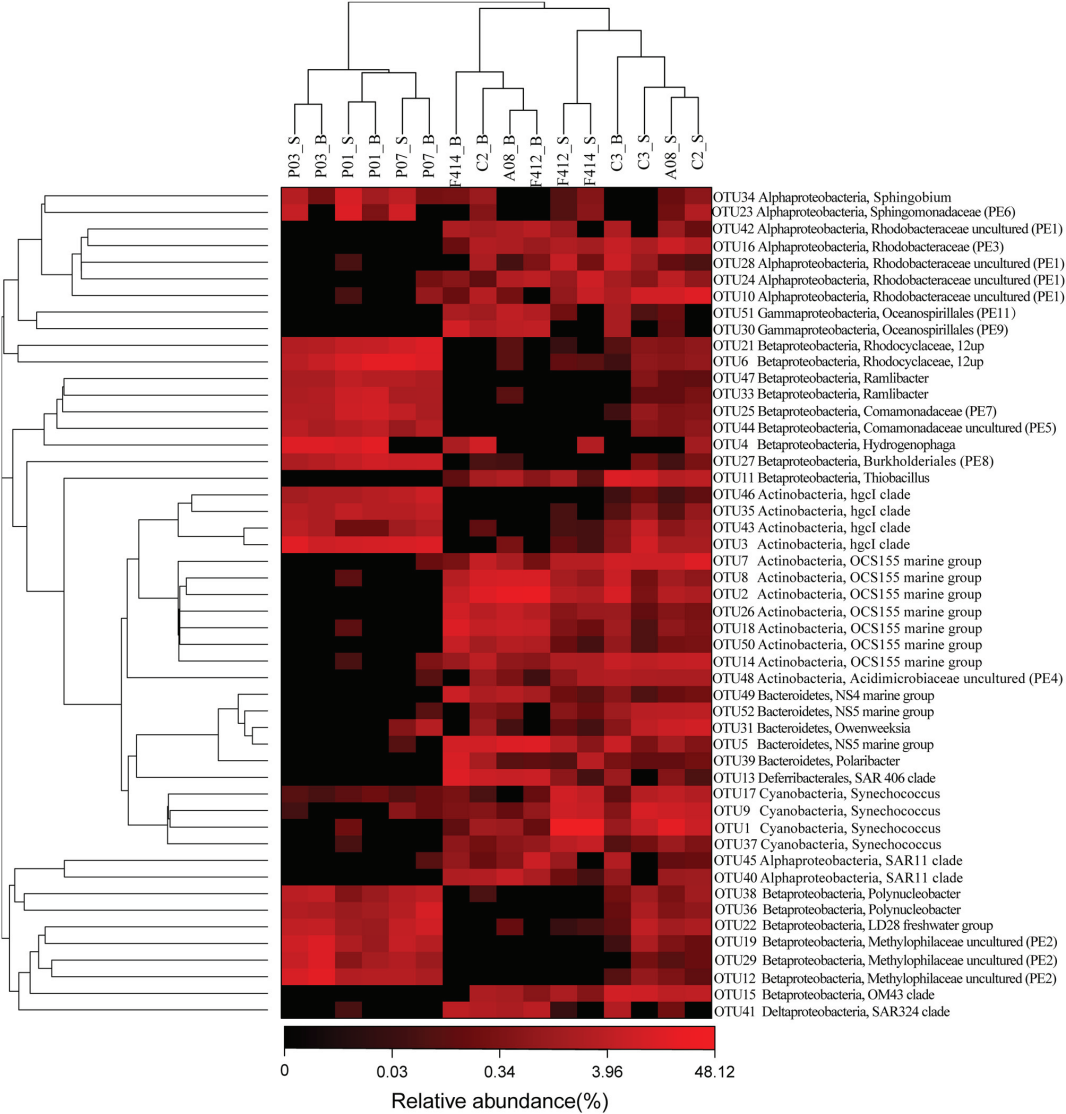
**The heatmap of the top 50 abundant OTUs ranked from OTU1 to OUT52 according to abundance**. OTU20 and OTU32 were removed because they belonged to chloroplast across all samples. Their phylogenetic relationships were shown on the right tree (constructed by FastTree using Maximum Likelihood method). The top tree showed the clustering relationship of samples. Eleven undefined clades were named as PE1-11, ranked by abundance. _S and _B standed for the surface and bottom water, respectively.

Eleven clades, either within the top 50 OTUs (see below) unaffiliated to any described genera in SILVA or the top 50 genera undesignated at a given family (for example, *Rhodobacteraceae* uncultured or *Acidimicrobiaceae* uncultured), were designated PE1-11 at the genus level, respectively (ranked by abundance, Table S2 and Figure 3).

**Figure 4:**
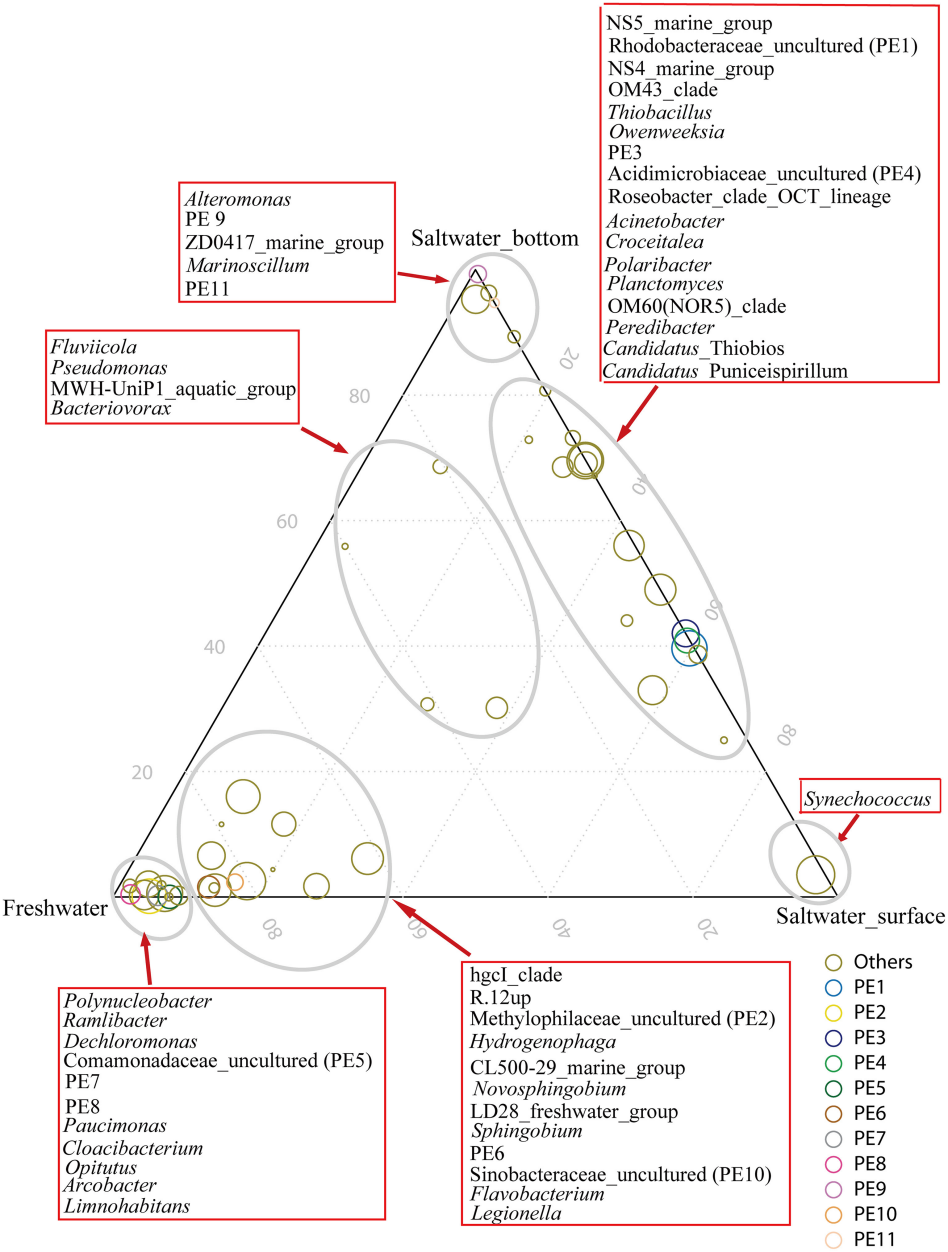
**Ternary plot generated in R software showing the distribution and their relative abundances of the top 50 genera in the freshwater sites, and surface water and bottom water of the saltwater sites**. The size of the symbol indicated relative abundance of each genus. Taxonomic affiliations were indicated by colors.

The hgcI clade (*Actinobacteria*), followed by R.12up (*Rhodocyclales*) and *Hydrogenophaga* (*Burkholderiales*), as well as the newly named PE6-8 clades, were abundant in the freshwater sites. In the saltwater sites, PE1 and PE3 clades were abundant at both depths with slightly higher proportions in the surface water. Meanwhile, *Synechococcus*, and PE9 and PE11 dominated the surface and bottom waters, respectively. Furthermore, the proportions of genera such as *Roseobacter* and *Thiobacillus* increased. Clades affiliated to *Flavobacteriaceae* shifted from *Cloacibacterium* in the freshwater sites to NS5, NS4 marine group, *Owenweeksia* and *Polaribacter* in the saltwater sites (Figure 4). *Fluviicola*, affiliated to *Bacteroidetes*, was one member of the very few genera that commonly distributed in the sampling areas (Figure 4). However, the freshwater-dominant members of *Fluviicola* formed a monophyletic cluster as shown in the phylogenetic tree constructed using sequences of the top 16 *Fluviicola* OTUs (Figure S3).

The bacterial community compositions of the hypoxic (P01 and P07) sites were distinguished from that of the non-hypoxic site (P03) (Figure S4). In concordance with the order level result, the abundance of PE2, a prevalent genus of *Methylophilaceae* in the freshwater sites, was significantly higher in the non-hypoxic sites than the hypoxic sites, whereas R.12up affiliated to *Rhodocyclales* showed an opposite trend. In addition, the hypoxic sites harbored more unclassified reads at both the order and genus levels.

### OTU level analysis and multi-sample comparison

While significant phylogenetic shifts were observed at the order level, OTU level analysis facilitated discerning reads even belonging to the same genus. The abundance of OTU 5 belonging to NS5 marine group was higher in the bottom water of saltwater sites while OTU 52, also affiliated to NS5 marine group, was more abundant in the surface water. Analogously, within the OCS155 marine group, OTU 7 and OTU 14 were more abundant in the surface water while OTU 2, 8, 18, 26, and 50 showed opposite trends (Figure 3).

**Figure 5:**
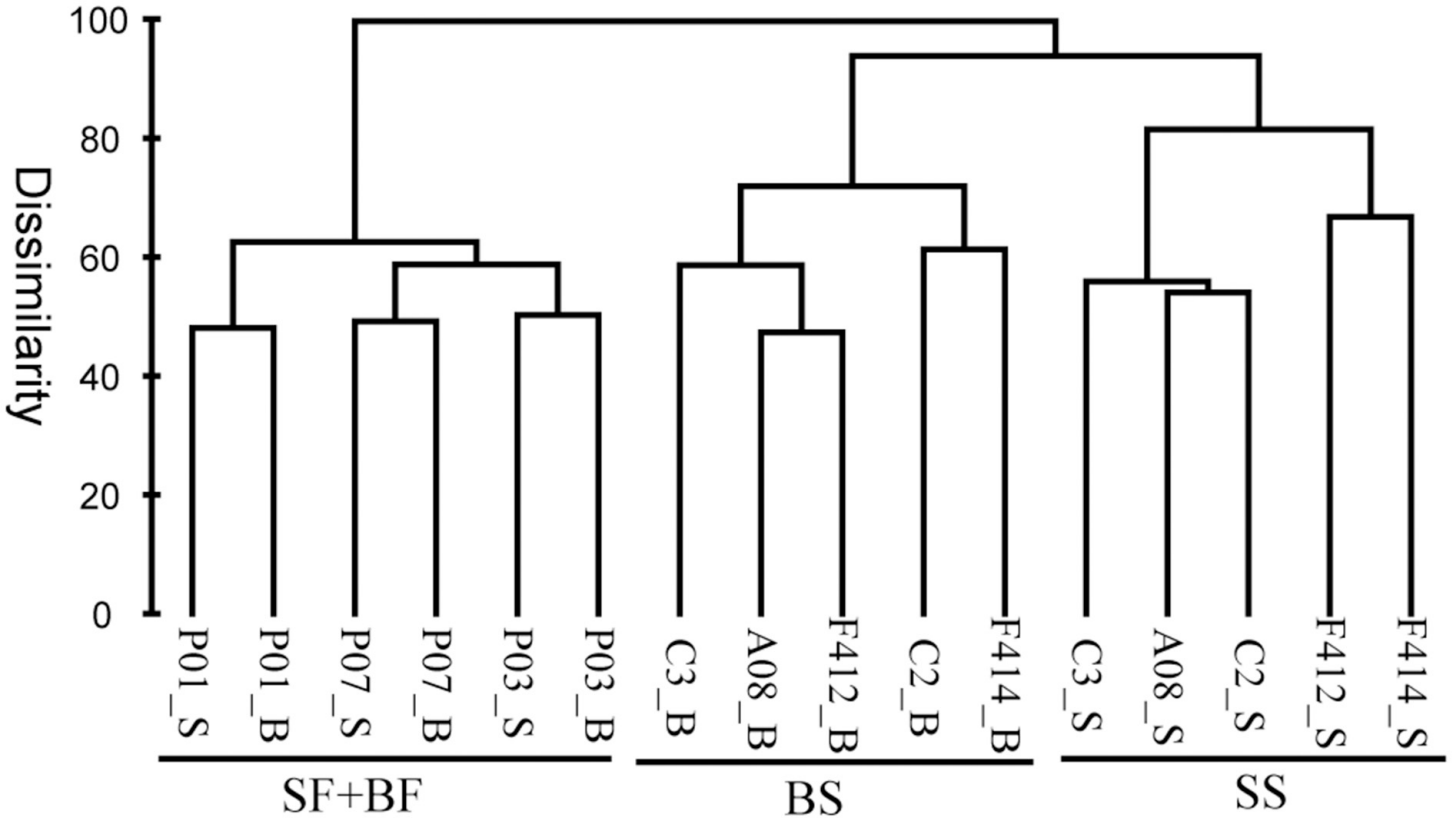
**Bray-Curtis dissimilarity based dendrogram showing the clustering of samples at the OTU level**. The hierarchical clustering was calculated with average linkage algorithms using PRIMER 5. SF, freshwater surface; BF, freshwater bottom; SS, saltwater surface; BS, saltwater bottom. _S and _B standed for the surface and bottom water, respectively.

Based on the composition and abundance of OTUs, the multi-sample dendrogram clustered all samples into three groups: surface and bottom waters of the freshwater sites (SF+BF), surface waters of the saltwater sites (SS), and bottom waters of the saltwater sites (BS) (Figure 5). The ANOSIM *R* statistic (Table S3) further confirmed the grouping result, as no significant variation was observed between SF and BF (*R* = −0.407, *P* > 0.05).

### The relationship between major bacterial clades and environmental factors

**Figure 6:**
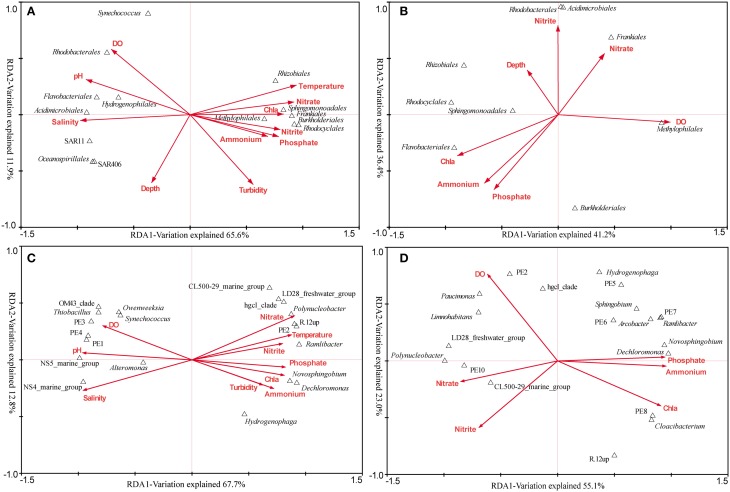
**The relationship between major bacterial taxa (taxonomy of sequences) and environmental factors. (A)** RDA across all samples at the order level. **(B)** RDA targeting only freshwater samples at the order level. **(C)** RDA across all samples at the genus level (the top 20). **(D)** RDA targeting only freshwater samples at the genus level (the top 20). In the RDA targeting freshwater sites, seven environmental factors were analyzed as the others were essentially the same.

The RDA across all samples was conducted to find the determinant environmental parameters shaping specific orders (Figure 6A). The first axis explained 65.6% of the total variance while the second axis explained 11.9%. Montel Carlo permutation tests showed that most of the environmental parameters except for depth significantly (*P* < 0.05) contributed to the heterogeneous distribution of major bacterial clades. Salinity explained most of the variation, and different clades of *Betaproteobacteria* and *Actinobacteria* were observed occupying distinct salinity ranges. N, P nutrients were also determinant factors. *Sphingomonadales* was found to be more related to NO^−^_3_, while *Rhodocyclales* and *Methylophilales* were positively related to NO^−^_2_ and PO^3−^_4_. In addition, *Synechococcus* was negatively related to turbidity, in agreement with the result obtained by flow cytometry.

The overall comparison would obscure the impact of some important factors, due to the complex environmental features of the Pearl Estuary. Therefore, RDA analysis targeting only freshwater sites was conducted, as quite different DO levels were observed among the three sites. DO (*F* = 2.76, *P* < 0.01) and Chl *a* (*F* = 2.51, *P* < 0.01) were the only two significant factors accounting for the variability. *Methylophilales* was positively correlated to DO, while *Rhodocyclales*, *Rhizobiales* and *Sphingomonadales* were on the opposite correlation (Figure 6B). Although not significant, *Frankiales* seemed to be positively correlated with NO^−^_3_, while negatively correlated with PO^3−^_4_ and NH^+^_4_. Taken together, these results were quite different from that revealed by the overall RDA, indicating a significant impact of hypoxia on bacterial structure.

In-depth genus distribution patterns determined by environmental factors revealed that many genera such as R.12up (*Rhodocyclales*), PE2 (*Methylophilales*) and hgcI (*Actinobacteria*) were inversely related to salinity. Nevertheless, PE2 and R.12up as well as *Novosphingobium* and *Dechloromonas* were also positively correlated to nutrients (Figure 6C). Specifically, *Novosphingobium* and *Dechloromonas* were positively correlated to PO^3−^_4_ and NH^+^_4_, while PE2 and R.12up were positively correlated to NO^−^_3_ and NO^−^_2_ (Figure 6C).

The RDA targeting only freshwater sites was also conducted at the genus level. We surprisingly found that NH^+^_4_, PO^3−^_4_, and NO^−^_3_, instead of DO, became the most significant environmental factors. *Polynucleobacter* and CL500-29 marine group were driven by NO^−^_3_/NO^−^_2_, while *Novosphingobium* and *Dechloromonas* were still correlated well with PO^3−^_4_ and NH^+^_4_. At the same time, PE2 was positively correlated with DO, whereas R.12up was on the opposite position (Figure 6D).

### Comparison of bacterial community between the pearl estuary and temperate estuaries

Bacterial community data of the Delaware Bay (Campbell and Kirchman, 2013) and Baltic Sea (Herlemann et al., 2011) were used as proxies of temperate estuaries. These two datasets were selected because they were both carried out using the high throughput sequencing and the PCR fragments were overlapped with that of this study. The bacterial community of the Baltic Sea was used because it receives large freshwater discharges which generate far-reaching saltwater-freshwater gradients, although not being a typical estuary. The phylogenetic tree (Figure S5) showed that *Verrucomicrobia* was a prevalent phylum in the two temperate estuaries, but accounted for only a minor proportion in the subtropical Pearl Estuary. SAR11 clade was found in all the three locations whereas *Sphingomonadales* and *Rhizobiales* were only detected in the Pearl Estuary, which might resulted from the high nutrient inputs. Moreover, among *Rhodobacterales*, sequences obtained from the Baltic Sea were phylogenetically different from that from the Pearl Estuary.

## Discussion

Bacterial community compositions in the Pearl Estuary and adjacent Hong Kong waters have previously been reported. These works have either provided microbial profiles at broad taxonomic levels or focused mainly on the surface water of the saltwater area (Wu et al., 2004; Zhang et al., 2006, 2007, 2009; Jing and Liu, 2012). However, a more resolved study targeting a finer taxonomic level can enhance our understanding of accurate microbial distribution patterns in response to environmental changes. Here, as a complement to previous studies, our study assessed both the horizontal and vertical distribution patterns of bacterioplankton communities as well as individual taxa along the Pearl Estuary. To our best knowledge, it is the first investigation regarding the impact of hypoxia on bacterioplankton communities in the upstream of the Pearl Estuary.

### Environment-dependent horizontal distribution of bacterioplankton community

Zhou et al. (2011) reported a decline trend of heterotrophic bacterial abundance from freshwater to coastal areas of the Pearl Estuary along with the decrease of nutrients. Our study confirmed this result as heterotrophic bacteria was found to be positively related to nutrients. Similar to previous results on estuaries, a significant variation in bacterioplankton community between freshwater and saltwater sites was observed (Kirchman et al., 2005; Zhang et al., 2006). However, a major finding of our study was the phylogenetic shifts in clades occupying different estuarine areas, consistent with the result in the Baltic Sea (Herlemann et al., 2011). *Alphaproteobacteria* was generally abundant in marine waters (Kirchman et al., 2005; Zhang et al., 2006). Nonetheless, we observed a relative small difference in the abundance of *Alphaproteobacteria* between the two areas of the Pearl Estuary, with *Rhodobacterales* (a common *Alphaproteobacteria* order in the polyhaline water, Campbell and Kirchman, 2013) dominating the saltwater sites while *Sphingomonadales* and *Rhizobiales* showing opposite distributions. *Sphingomonadales* has been documented to have wide metabolic capabilities (Miller et al., 2010) and can degrade aromatic compounds (Fredrickson et al., 1995), and its abundance, especially that of *Novosphingobium* and *Sphingobium*, appeared to be driven by high concentrations of PO^3−^_4_ and NH^+^_4_ (Figure 6). Additionally, *Rhizobiales* was active in biofilm formation under both oxic and anoxic conditions (Masuda et al., 2010). Thus, the prevalence of them in the freshwater area contributed to the comparable abundance of *Alphaproteobacteria* between the two end members of the Pearl Estuary and might be attributed to the highly polluted condition resulted from the increasing anthropogenic disturbances. These results indicated that the high concentration of nutrients has shifted the bacterial community components toward copiotrophic groups.

Among *Actinobacteria*, OCS155 marine group and hgcI were the two groups dominant in the saltwater and freshwater areas, respectively. Since its first discovery in Oregon coastal waters (Rappe et al., 2000), the OCS155 marine group has been found in various marine waters (e.g., Lu et al., 2015), but its function is still unknown. The hgcI clade, also known as acI, is common and abundant in a wide range of freshwater habitats (Warnecke et al., 2004), a recent single-cell genomic study showed that it had a strong genetic ability to take carbohydrate and N-rich organic compounds (Ghylin et al., 2014). The hgcI clade had also the potential to utilize sunlight via actinorhodopsin which might promote anaplerotic carbon fixation (Ghylin et al., 2014), indicative of both heterotrophic and autotrophic lifestyles of this clade. In this study, although the abundance of hgcI clade was constant in hypoxic and non-hypoxic samples, no apparent phylogenetic shift between these sites was observed (data not shown). This suggests that the hgcI clade could tolerant to a lower DO content and the maintaining mechanism needs to be investigated further.

It was interesting that *Thiobacillus*, affiliated to *Hydrogenophilales* of *Betaproteobacteria* was found prevalent in the saltwater area of the Pearl Estuary, different from the classical freshwater *Betaproteobacteria* such as *Rhodocyclales* and *Burkholderiales*. *Thiobacillus* is a well-known sulfur-oxidizing bacteria and its predominance may suggest frequent occurrence of sulfur oxidization process in the estuarine water. Additionally, some genera of *Betaproteobacteria* were driven by nutrients, for example, *Dechloromonas* of *Rhodocyclales* was positively correlated with PO^3−^_4_ in both RDAs (Figures 6C,D). The extent to which clades of *Betaproteobacteria* in freshwater systems are driven by environmental variables such as nutrients worths further investigation. Furthermore, among the several genera that distributed commonly in the sampling areas (Figure 4), members of *Fluviicola* displayed a clear phylogenetic shift from the freshwater to saltwater sites. Our results suggest the presence of distinct *Fluviicola* phylotypes in saltwater environments from so far isolated species, which grow in the absence of sodium ion (O'Sullivan et al., 2005; Yang et al., 2014). Similarly, Luo et al. (2014) has demonstrated that the isolated and yet uncultivable *Roseobacters* had clearly distinct genetic characters. Taken together, in-depth taxonomic assignments can reveal a more resolved microbial distribution pattern, which would provide insight into the ecology of bacteria.

### Environment-dependent vertical distribution of bacterioplankton community

Although the average water depth of the Pearl Estuary was shallower (<50 m), a clear stratified variation in bacterioplankton community in the saltwater sites was observed, compared with the consistency of bacterioplankton community in the two water layers of the freshwater sites. Previous studies have reported a significant depth-related bacterial variability in estuarine plumes with a deeper water depth, where the actual influential factor resulted in this variance is elusive (Herlemann et al., 2011; Fortunato et al., 2012). In the Pearl Estuary, the difference in water masses with different chemical parameters might be one plausible interpretation for this disparity as marine water enters the estuary at the bottom and freshwater flows out of the estuary at the surface (Dong et al., 2004).

The predominance of *Synechococcus* in the downstream surface of the Pearl Estuary was consistent with a previous study on the Baltic Sea where light attenuation might be the determining factor (Herlemann et al., 2011). Despite the shallow water depth of the Pearl Estuary, the high turbidity in the bottom water of saltwater sites would prevent penetration of light into the bottom and thus inhibit the growth of *Synechococcus*. Williams et al. (2012) demonstrated a nutrient cycle in the surface water of the coastal East Antarctica, where *Flavobacteria*-mediated algal organic matter degradation could facilitate the growth of SAR11 clade and marine *Gammaproteobacteria* (mainly *Oceanospirillales* and *Alteromonadales*). Here, high abundance of picoplankton (both prokaryotic and eukaryotic), and SAR11 clade and *Gammaproteobacteria* was observed in surface and bottom waters of the saltwater sites, respectively. As marine water enters the estuary bottom during summer (Dong et al., 2004), it is likely that both SAR11 clade and *Gammaproteobacteria* (mainly *Oceanospirillales* in this study) are of ocean origin and come from the surface water of the South China Sea. This, together with the existence of *Flavobacteria* in the bottom water, led to the hypothesis that nutrients cycle using organic matter from the ocean or sinking from the surface to the bottom was prevalent in the bottom water of the Pearl Estuary.

### The influence of hypoxia on bacterioplankton community

Hypoxia is becoming one of the most serious problems in various ecosystems, and its impact on bacterial assemblages has been documented in many marine and fresh water systems (Stevens and Ulloa, 2008; Zaikova et al., 2010; Li et al., 2012; Peura et al., 2012) but rare in estuarine areas (Crump et al., 2007; Hewson et al., 2014). Microbes can alternatively use a variety of terminal electron acceptors, which enables their growth under various oxygen regimes (Eggleston et al., 2014; Hewson et al., 2014). Concomitantly, phylogenetic and functional transitions of bacterial community under different oxygen conditions have been observed in mesohaline waters of the Chesapeake Bay, and the most extensive shifts occurred during onset of hypoxic condition (Hewson et al., 2014). In the present study, the seasonal hypoxia in the bottom water of the lower Pearl Estuary was not observed, in part due to the effect of typhoon just before of our cruise. However, whole water column hypoxia, mainly induced by high organic matter and nutrient loads, was observed in two freshwater sites (P01 and P07) of the Pearl Estuary, similar to that reported by Dai et al. (2006). It was notable that the DO level of P03, in the midway of P01 and P07, was unexpectedly higher, but the mechanism and the existing time of this were uncertain. Although with a relatively few number of samples, DO accounted for most of the bacterioplankton variation in the RDA targeting only freshwater sites, remarkably different from that targeting all samples. It might be the overwhelming salinity that obscured the impact of DO in the local region. However, the most significant environmental factor in governing bacterial assemblages in the RDA targeting only freshwater sites shifted from DO (at the order level) to nutrients (at the genus level). Close correlations of some specific genera with nutrients such as *Novosphingobium* and *Sphingobium* might have diluted the effect of DO.

*Rhodobacterales* and *Methylophilales* (mainly PE2) were positively correlated to DO in the RDA targeting all samples and only freshwater sites (Figure 6), respectively. *Methylophilales* has been reported to be involved in the process of methylotropic metabolism (Lueders et al., 2004), and it is dominant in oxic surface waters (Wright et al., 2012) such as the Dongjiang River, another tributary of the Pearl Estuary with DO level >3 mg L^−1^ (Liu et al., 2012), suggesting that oxygen is requisite for the methylotropic metabolism in the Pearl River. On the other hand, the abundances of *Rhodocyclales* (mainly R.12up), *Sphingomonadales* and *Rhizobiales* increased when oxygen was depleted. *Rhodocyclales* has been demonstrated its capability of pollutants degradation (Loy et al., 2005), and the phylogenetic relative of R.12up, “*Candidatus Accumulibacter*,” was reported to involve in the phosphorus removal from wastewater (Tsuneda et al., 2005). The prevalence of R.12up, along with *Sphingomonadales* and *Rhizobiales* as mentioned above, reflected the nearly anoxic and highly polluted condition of the Pearl Estuary. *Burkholderiales*, the most abundant order in the freshwater sites of the Pearl Estuary, as well as “*Candidatus Accumulibacter*” was reported to be involved in denitrification (Saito et al., 2008; Hesselsoe et al., 2009). Contrasting to the higher ratio of denitrifier, a relatively lower ratio of nitrifier was observed in our study. However, nitrification has been demonstrated to contribute substantially to the consumption of DO in the upper reach of the Pearl Estuary (Dai et al., 2006, 2008) in addition to aerobic respiration. The lower abundance of nitrifier might indicate a greater nitrification rate per cell in the wet season as suggested by Dai et al. (2008). Another explanation for the oxygen depletion might be attributed to the high abundance of Marine Group I, a widely known aerobic ammonia-oxidizing archaea (Mußmann et al., 2011), in the freshwater sites of the Pearl Estuary (Liu et al., 2014). Additionally, phylogenetically different Marine Group I subclades were observed between the hypoxic and non-hypoxic water samples (Liu et al., 2014). The nitrification capability of the hypoxia-adapted Marine Group I subclade is unknown and worths further investigation. In all, these results demonstrated substantial metabolic shifts between the hypoxic and non-hypoxic sites in the upper reach of the Pearl Estuary.

### Comparison of bacterial community between the pearl estuary and temperate estuaries

Although microorganisms have a cosmopolitan distribution, rare and extreme environments (e.g., hot springs) have been found to possess some special ecotypes. As a consequence, it is interesting to investigate the bacteria inhabiting geographically distant non-extreme environments. Different climate zones therefore provide a natural geographical segmentation for such surveys. The phylogenetic tree (Figure S5) showed that the Pearl Estuary harbored fundamentally different bacterial communities from the temperate Delaware Bay (Campbell and Kirchman, 2013) and Baltic Sea (Herlemann et al., 2011). It was notable that *Sphingomonadales* and *Rhizobiales* of *Alphaproteobacteria* were only detected in the Pearl Estuary. As mentioned above, the high nutrient loads might favor the growth of these groups, which might indicate more intensive anthropogenic activities compared with the other estuaries. In addition, *Verrucomicrobia* was prevalent in the two temperate estuaries, which on the contrary made up only a minor proportion in the Pearl Estuary. The high nutrients concentration sustained a high biomass of phytoplankton, consistent with the high Chl *a* in the water column of the Pearl River, and the high abundance of phytoplankton might reduce the availability of P for the bacterioplankton (Lindström et al., 2004). Thus, the high abundance of phytoplankton might have inhibited the growth of *Verrucomicrobia* in the Pearl River. Moreover, among *Rhodobacterales*, sequences obtained from the Baltic Sea were phylogenetically different from that from the Pearl Estuary. Despite that evident differences were observed between bacterial communities of different climate zones, to what extent temperature and other environmental factors contributed to the variation should be further solved.

## Conclusion

The present study provided a detailed comparison of bacterioplankton community and specific taxa in freshwater and saltwater regions of the Pearl Estuary using high-throughput sequencing, and remarkable differences between the two areas were observed with clear phylogenetic shifts in bacterial clades within the same taxa. Meanwhile, the depth related variation was evident in the shallow saltwater sites, which was attributed to the two different water mass sources and light availability (turbidity dependent). Eleven clades were designated, which might reflect the unequaled ecological value of the Pearl Estuary. The RDA analysis targeting all samples or only the freshwater sites provided quite distinct results. Different DO and nutrient levels were observed in the freshwater areas which significantly accounted for changes of bacterioplankton communities. However, across all samples, salinity and nutrients were determinant factors that partitioned the two regions, and the role of hypoxia has been obscured by the overwhelming impact of these two factors in the overall analysis.

### Conflict of interest statement

The authors declare that the research was conducted in the absence of any commercial or financial relationships that could be construed as a potential conflict of interest.
