# Supplementary material for: Phylogenetic shifts of bacterioplankton community composition along the Pearl Estuary: the potential impact of hypoxia and nutrients
Source: Front Microbiol. 2015 Feb 10;6:64. doi: 10.3389/fmicb.2015.00064 (PMC4322608; doi:10.3389/fmicb.2015.00064)
Supplement: Supplementary file 1 [file Presentation1.PDF]

Phylogenetic shifts of bacterioplankton community composition along the Pearl Estuary: the potential impact of hypoxia and nutrients

Jiwen Liu, Bingbing Fu, Hongmei Yang, Meixun Zhao, Biyan He and Xiao-Hua Zhang

Supporting Information

Table S1 Environmental parameters of the 16 water samples. _S and _B stands for the surface and bottom water, respectively

Samples	Depth<br>m	Temperature<br>℃	Salinity<br>PSU	pH	Turbidity<br>FTU	DO<br>mg L^{-1}	Chl *a*<br>μg L^{-1}	PO_4^{3-}<br>μmol L^{-1}	NO_3^-<br>μmol L^{-1}	NO_2^-<br>μmol L^{-1}	NH_4^+<br>μmol L^{-1}
P01_S[a]	1	30.14	0.00	7.01	24.10	0.22	16.45	4.8	78.6	14.8	139.7
P01_B[a]	7	30.10	0.00	7.01	24.11	0.51	17.36	4.7	96.1	14.7	140.1
P03_S[a]	1	29.61	0.00	7.24	24.10	3.47	5.16	2.0	102.5	24.6	21.4
P03_B[a]	3	29.61	0.00	7.21	24.08	3.14	6.45	2.2	103.0	26.4	30.6
P07_S[a]	1	29.35	0.00	6.99	22.14	0.72	11.54	2.0	105.0	43.1	34.4
P07_B[a]	19	29.32	0.10	6.98	24.11	0.62	9.73	2.0	106.4	41.9	31.1
C2_S[a]	1	28.31	18.66	8.01	9.08	5.48	1.55	1.0	54.3	6.5	1.1
C2_B[a]	6	27.22	28.34	8.03	24.11	4.09	1.13	0.8	18.5	4.9	n.d.
C3_S	1	28.54	7.84	7.99	n.a.	6.82	1.10	1.5	92.2	7.3	8.2
C3_B	6	26.47	25.44	7.98	16.23	4.89	0.16	1.0	22.6	3.3	n.d.
A08_S[a]	1	28.34	16.50	8.04	5.26	6.45	4.81	1.0	61.5	8.5	0.8
A08_B[a]	13	26.45	31.50	8.09	23.42	4.68	0.91	0.5	4.4	4.6	n.d.
F412_S[a]	1	27.50	23.50	8.11	3.90	6.95	4.29	0.9	36.9	4.2	n.d.
F412_B[a]	14	26.39	31.41	8.13	7.82	5.39	n.a.	0.4	4.4	1.8	n.d.
F414_S	1	28.61	23.10	8.24	3.42	7.59	4.46	0.2	35.6	4.5	n.d.
F414_B	27	25.36	33.60	8.07	4.24	3.85	n.a.	0.4	3.1	1.0	0.9

Abbreviation: DO, dissolved oxygen; PSU, practial salintiy units; FTU, Formazin turbidity units.

n.a., no analysis; n.d., non detectable.

[a], Environmental factors of these samples have been reported in Liu et al. (2014).

Table S2. The relative frequency of the most abundant 50 genera or equivalent-level clades (with the 11 newly named clades) across all sample among the three groups (ranked by abundance in 16 samples).

Taxonomic group	Freshwater %	SS %	BS %
Synechococcus	0.260	22.664	0.762
hgcI_clade	10.259	3.128	0.423
NS5_marine_group	0.008	3.110	6.348
R.12up	6.948	0.657	0.046
PE1 (Rhodobacteraceae_uncultured)	0.050	5.655	2.935
PE2 (Methylophilaceae_uncultured)	6.676	0.459	0.014
Hydrogenophaga	3.340	0.616	0.965
NS4_marine_group	0	1.465	3.041
Polynucleobacter	2.936	0.675	0.023
CL500-29_marine_group	1.971	1.452	0.156
OM43_clade	0	2.141	1.658
Thiobacillus	0.027	1.764	1.699
Ramlibacter	2.626	0.156	0.005
Owenweeksia	0.279	1.902	0.909
Alteromonas	0.046	0.060	2.457
Novosphingobium	0	1.383	0.896
PE3	1.416	0.276	0.133
Dechloromonas	1.493	0.083	0.037
LD28_freshwater_group	1.359	0.666	0.030
PE4 (Acidimicrobiaceae_uncultured)	0	1.346	0.753
Sphingobium	1.148	0.331	0.248
PE5 (Comamonadaceae_uncultured)	1.431	0.147	0
Roseobacter_clade_OCT_lineage	0.004	0.556	1.079
PE6	1.412	0.271	0.018
Fluviicola	0.489	0.721	0.587
Acinetobacter	0.049	0.519	1.088
PE7	1.516	0.133	0.004
PE8	1.435	0.041	0.009
Paucimonas	1.202	0.165	0.005
Croceitalea	0	1.047	0.482
PE9	0	0.014	1.488
PE10 (Sinobacteraceae_uncultured)	1.137	0.289	0.027
ZD0417_marine_group	0	0.037	1.240
Polaribacter	0	0.303	0.928
Pseudomonas	0.188	0.133	0.785
Cloacibacterium	0.758	0.023	0.018
MWH-UniP1_aquatic_group	0.302	0.395	0.298
Marinoscillum	0	0.101	1.034

Planctomyces	0.061	0.560	0.413
OM60(NOR5)_clade	0	0.229	0.818
Opitutus	0.685	0.156	0.009
PE11	0	0.064	0.909
Arcobacter	0.704	0.064	0.018
Limnohabitans	0.731	0.083	0
Peredibacter	0.061	0.239	0.501
*Candidatus*_Thiobios	0.019	0.735	0.152
Bacteriovorax	0.291	0.032	0.542
*Candidatus*_Puniceispirillum	0	0.229	0.418
Flavobacterium	0.448	0.060	0.096
Legionella	0.498	0.175	0.013

Abbreviation: SS, surface water of saltwater sites; BS, bottom water of saltwater sites

Table S3. Pairwise comparison of similarity (ANOSIM) among bacterial communities.

Groups	SF	BF	SS	BS
SF				
BF	0.407, $P > 0.05$			
SS	0.877, $P < 0.05$	0.887, $P < 0.05$		
BS	1, $P < 0.05$	1, $P < 0.05$	0.696, $P < 0.05$	

Abbreviation: SF, freshwater surface; BF, freshwater bottom; SS, saltwater surface; BS, saltwater bottom

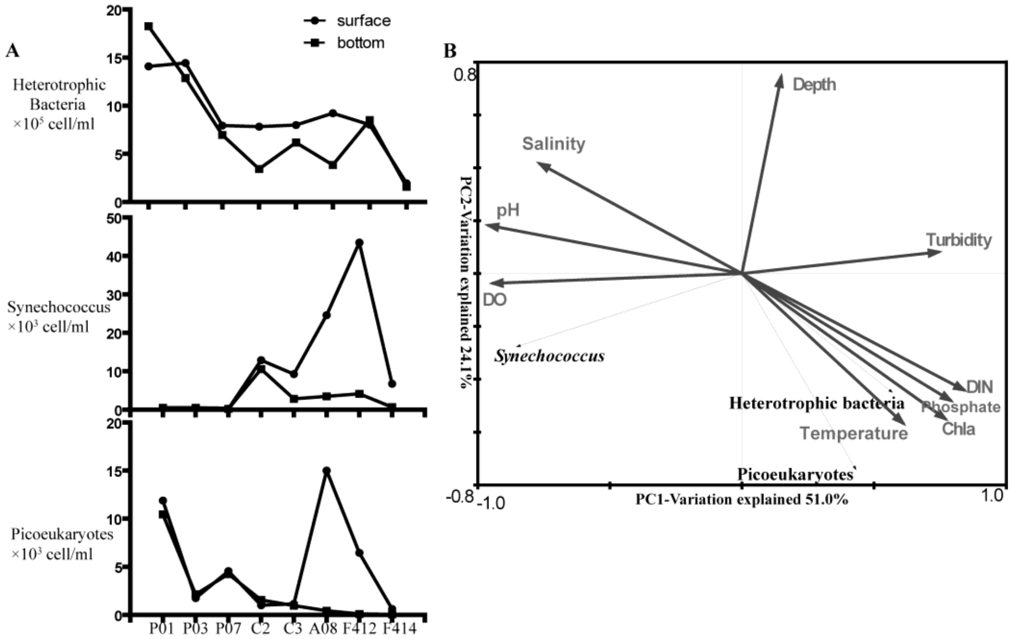

Figure S1. A: Abundance of heterotrophic bacteria, *Synechococcus* and picoeukaryotes across all samples. B: Distribution of picoplankton groups according to environmental factors by RDA.

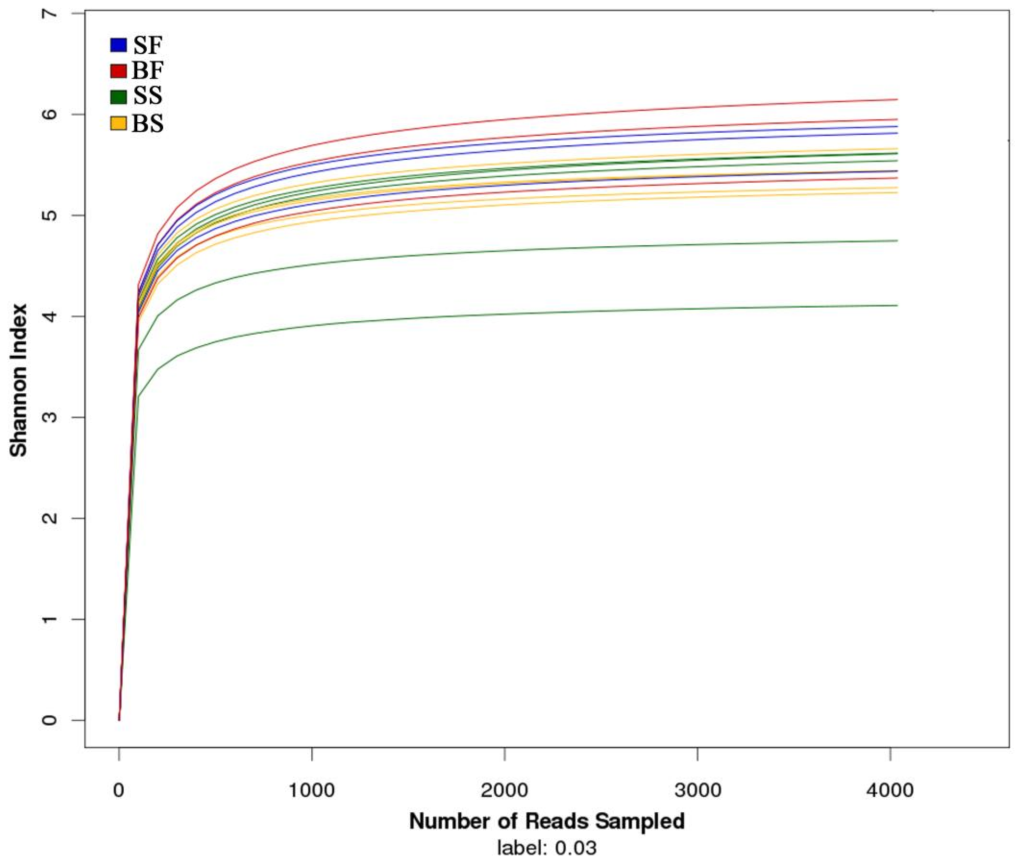

Figure S2. The Shannon index curves against sequence numbers which were plotted by normalized data set. Abbreviation: SF, freshwater surface; BF, freshwater bottom; SS, saltwater surface; BS, saltwater bottom.

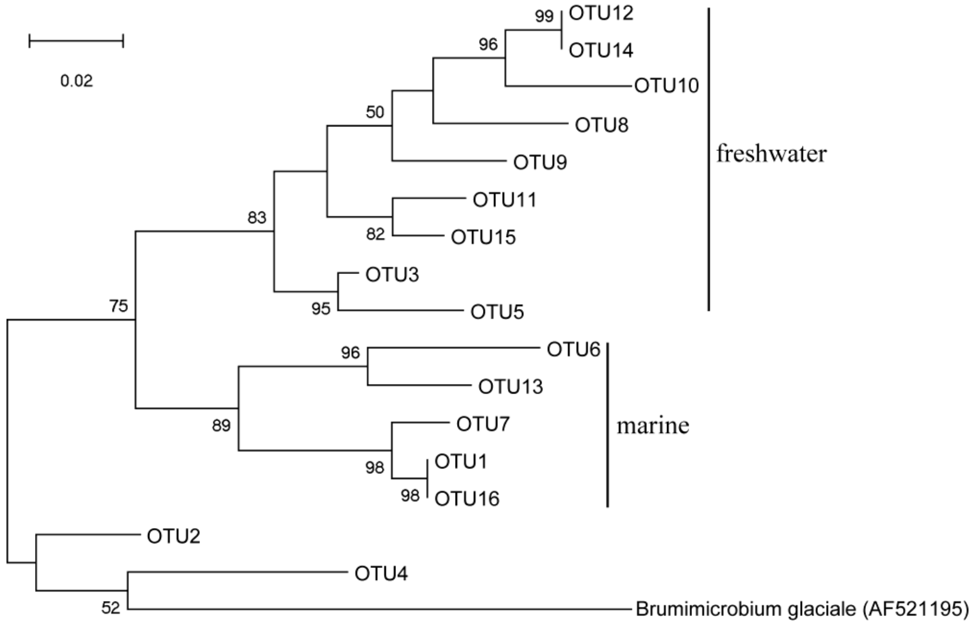

Figure S3. A maximum-likelihood phylogenetic tree constructed in MEGA 5 using sequences of the top 16 OTUs belonging to *Fluviicola* from this study showing that the freshwater- and marine- dominant clusters were phylogenetically different. The 16S rRNA sequence of *Brumimicrobium glaciale* was used as an outgroup.

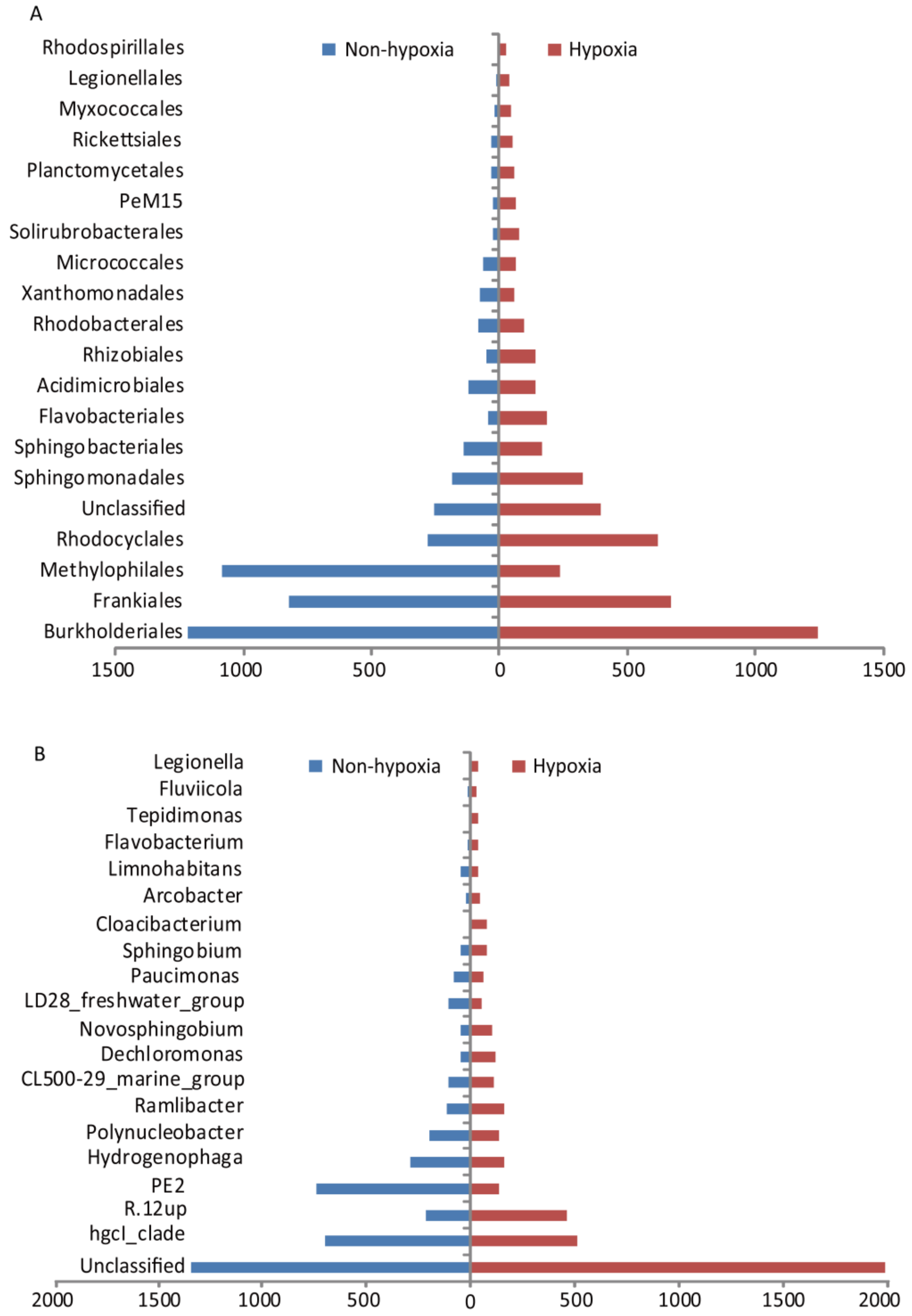

Figure S4. Comparison of read numbers between hypoxic and non-hypoxic sites of the freshwater sites at both the order (A) and genus (B) levels.

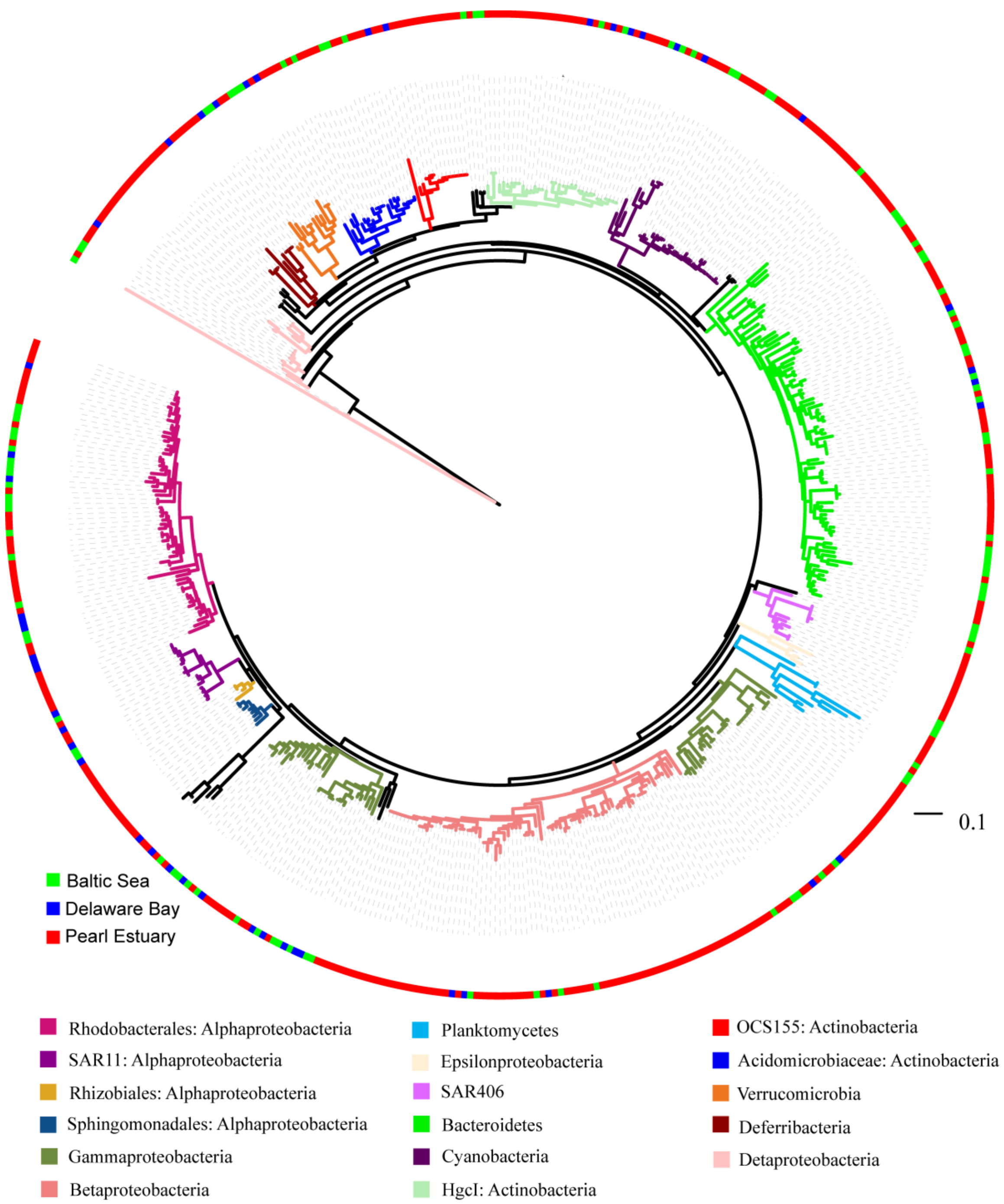

Figure S5. A neighbor-joining phylogenetic tree constructed using sequences of the top 50 OTUs of each samples from this study, and the top OTUs from the Delaware Bay (Campbell and Kirchman, 2013) and Baltic Sea (Herlemann *et al*., 2011).

Reference

Liu, J., Yu, S., Zhao, M., He, B., and Zhang, X.H. (2014). Shifts in archaeaplankton community structure along ecological gradients of Pearl Estuary. FEMS. Microbiol. Ecol. 90, 424-435. doi: 10.1111/1574-6941.12404.
